# A Multilayer Fusion Light-Head Detector for SAR Ship Detection

**DOI:** 10.3390/s19051124

**Published:** 2019-03-05

**Authors:** Yunchuan Gui, Xiuhe Li, Lei Xue

**Affiliations:** School of Electronics Contermeasure, National University of Defense Technology, No. 460, Huangshan Road, Shushan District, Hefei 230037, China; kwrgyc@gmail.com (Y.G.); guibs0821@163.com (L.X.)

**Keywords:** SAR ship detection, deep learning, multilayer fusion, light-head detector

## Abstract

Synthetic aperture radar (SAR) ship detection is a heated and challenging problem. Traditional methods are based on hand-crafted feature extraction or limited shallow-learning features representation. Recently, with the excellent ability of feature representation, deep neural networks such as faster region based convolution neural network (FRCN) have shown great performance in object detection tasks. However, several challenges limit the applications of FRCN in SAR ship detection: (1) FRCN with a fixed receptive field cannot match the scale variability of multiscale SAR ship objects, and the performance degrade when the objects are small; (2) as a two-stage detector, FRCN performs an intensive computation and leads to low-speed detection; (3) when the background is complex, the imbalance of easy and hard examples will lead to a high false detection. To tackle the above issues, we design a multilayer fusion light-head detector (MFLHD) for SAR ship detection. Instead of using a single feature map, shallow high-resolution and deep semantic feature are combined to produce region proposal. In detection subnetwork, we propose a light-head detector with large-kernel separable convolution and position sensitive pooling to improve the detection speed. In addition, we adapt focal loss to loss function and training more hard examples to reduce the false alarm. Extensive experiments on SAR ship detection dataset (SSDD) show that the proposed method achieves superior performance in SAR ship detection both in accuracy and speed.

## 1. Introduction

Synthetic aperture radar (SAR) is a coherent imaging technology that provides high-resolution, all-day, and all-weather images [1,2]. As a benefit from spaceborne SAR like Sentinel-1 [3], TerraSAR-X [4], and RADARSAT-2 [5], large volumes of high resolution SAR images are available. SAR ship detection, being a fundamental but challenging problem, has recently attracted considerable attention for its use in practical civil and military domains.

The task of object detection is to determine whether or not a given image contains objects of interest and locate the position of each predicted object in the image. Many investigations related to SAR ship detection have been carried out. Traditional SAR ship detection methods can be divided into statistically based and physically based methods. In statistically based methods, two-parameter constant false alarm rate (CFAR) [6] and its variations [7,8] are most widely used. Wan et al. [7] proposed an intensity-space (IS) domain CFAR ship detector. Image is transformed into a new IS domain and targets with high index pixels will be considered as ships. Li et al. [8] proposed an improved superpixel-level CFAR detection method by using the weighted information entropy (WIE) to describe the statistical characteristics of superpixel, yielding a better distinction between target and clutter superpixel. However, methods based on CFAR require high contrast between the target and background clutter in the SAR image, and it is based on the assumption that the statistical distribution model of background clutter is a Gaussian distribution. Besides, the fixed window size cannot suit the multiscale ship target. These detection methods work well in simple scenarios but get worse in complex situations. In physically based methods, Gambardella et al. [9] proposed a new physical approach, which considered ships as dominant scatterers and responsible for a strong and coherent backscattered signal. Jiang et al. [10] proposed ship detection based on the feature confidence, the features include kernel density estimation, length-width ratio, and the number of target pixels. Targets with high feature confidence will be interpreted as ships. However, the existence of SAR speckle noise makes it difficult to extract effective features for discrimination. Therefore, in order to obtain better SAR ship detection performance, it is necessary to develop a detector with strong feature extraction ability.

Deep learning is an automatic feature representation framework, which can learn deep features from the data itself. Owing to the rapid development of large-scale image datasets and graphics processing units (GPUs), convolution neural networks (CNNs), which are capable of hierarchical feature representation, have achieved prominent success in many computer vision tasks such as image classification, object detection, and image segmentation [11]. As object region is usually carried out from feature space, powerful feature representation is very important for constructing a high-performance detector. Object detection algorithms based on deep learning can be categorized into two-stage detectors and one-stage detectors. Two-stage detectors, represented by R-CNN series [12,13,14], have the advantage of higher detection. On the other side, one-stage detectors such as YOLO [15,16] and SSD [17] perform much faster than two-stage detectors while compromising accuracy, and they fall short when dealing with small objects. The demerit of one-stage detectors limits their application for SAR ship detection, therefore, we utilize two-stage method in our framework.

Benefiting from amazing breakthroughs and innovative structure, SAR ship detection based on deep CNNs has also been extensively studied during the past years. Li et al. [18] proposed a new dataset and several strategies such as feature fusion, transfer learning, and hard negative mining to improve the standard faster region based convolution neural network (FRCN) algorithm. Zhong et al. [19] proposed a multiscale object proposal network to generate region from different layers and regions of interest (RoIs) are taken from fused feature maps to enable small and densely packed objects to produce stronger response. Miao Kang et al. [20] presented a small sized ships detection framework which fuses the deep semantic and shallow high-resolution features, taking the additional contextual features to provide complementary information for classification and help to rule out false alarms. Jiao Jiao et al. [21] proposed a densely connected neural network based on FRCN to achieve multiscale and multi-scene SAR ship detection.

Due to the different characteristics of aerial view [22], the variable size of objects, and complex background scenes, directly applying deep learning detection methods cannot exhibit good performance in SAR ship detection. Comparing to natural images, in SAR ship detection it is more difficult to learn and extract representative features to distinguish them from other objects, especially for those small objects with several pixels. Besides, the additional multilayer combination will put heavy weight on the head of the network. Moreover, the dominance of easy examples during training makes it difficult for the detector to detect hard examples, and leads to a high false detection. To address these issues, inspired by [23], we propose a multilayer fusion light-head detector to detect multiscale objects. As a two-stage detector, the proposed method consists of three subnetworks: backbone network, region proposal subnetwork, and light-head detection subnetwork. We take ResNet as the backbone network for it is substantially a deep neural network and can ease the training process. To realize multiscale SAR ship detection, the proposed method fuses the shallow high-resolution and deep semantic features to generate region proposal. In order to improve the detection speed, in detection subnetwork, we adapt light-head design with large-kernel separable convolution and position-sensitive pooling layer. For the imbalance of easy and hard examples during the training process, the focal loss function is used to substitute for conventional cross entropy. Experiments on SAR ship detection dataset (SSDD) [18] prove that the proposed method achieves superior performance on detection accuracy and significantly improves the detection speed.

The rest of this paper is organized as follows. Section 2 states the details of the proposed method. Section 3 introduces the SAR image dataset and describes the experimental results to validate the effectiveness of the proposed method. Finally, the conclusions are drawn in Section 4.

## 2. Proposed Method

**Figure 1:**
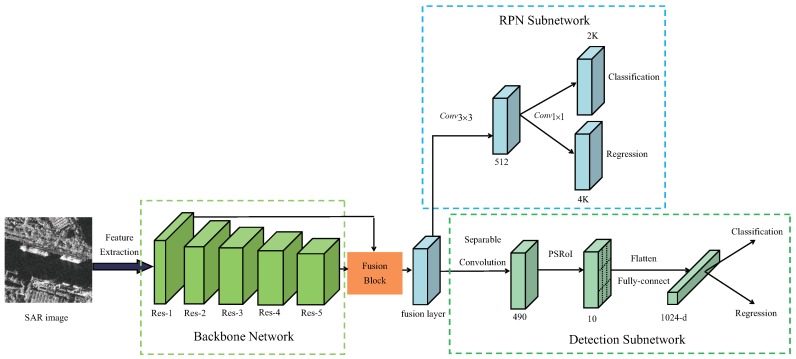
The architecture of proposed method.

An overview of the proposed framework is illustrated in Figure 1, the proposed network consists of three subnetworks, namely backbone network, RPN subnetwork, and detection subnetwork. The aim of the backbone network is to extract features from the original image and share the feature maps with the following two subnetworks. Next, a fusion block combine shallow/deep layer and output a fusion layer, the RPN subnetwork works to generate multiscale region proposals based on the fusion layer. Finally, the region proposals are sent to the detection subnetwork for accurate classification and regression. In this section, we will describe the design in details.

### 2.1. Backbone Network

**Figure 2:**
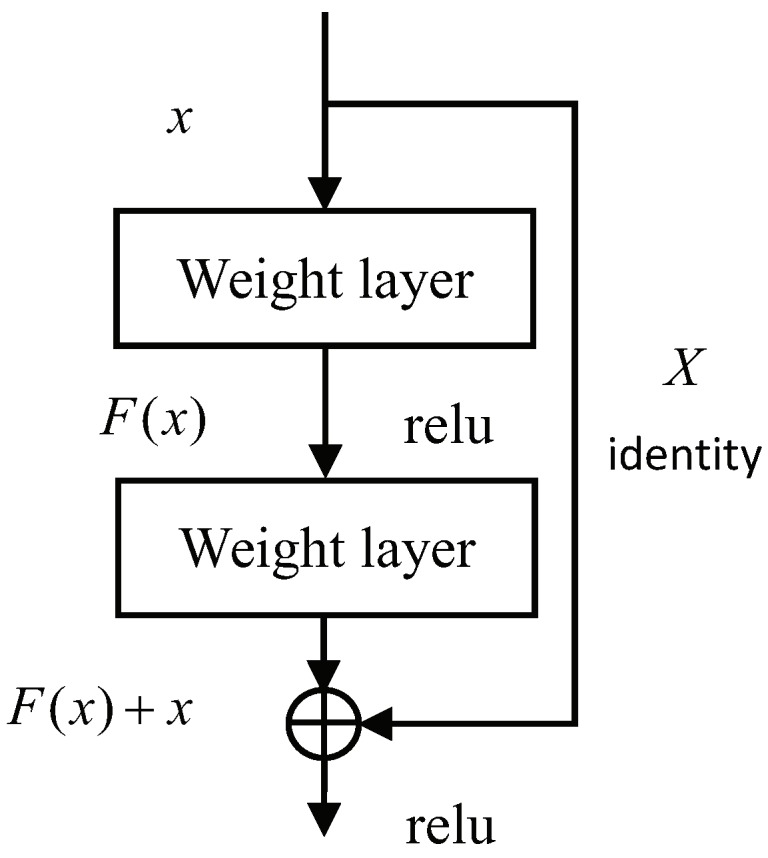
The shortcut connection.

The backbone network takes an image as input and outputs multiple level feature maps. It is noted that the depth of CNNs is very important to improve the performance of feature representation. However, with increasing depth, the network is more difficult to train for the reason of parameters explosion and gradient vanishing. Considering that deep neural networks are more difficult to train, ResNet [24] was proposed to adapt a residual learning framework to ease the training process. Instead of stacking convolution layers directly, ResNet connects these layers to fit a residual mapping. Denoting the input as *x* and the desired underlying mapping as H(x), we let the stacked nonlinear layers fit another mapping of F(x):=H(x)-x, then the original mapping is recast into F(x)+x, and the formulation can be realized by feedforward networks with shortcut connections as Figure 2. Shortcut connections add neither extra parameter nor computational complexity, and the entire network can propagate the signals with more layers by this strategy.

As a fully convolutional structure, the residual learning framework helps to improve the network depth and makes highly semantic feature representation possible. For simplicity and practicality, the main structures of ResNet have been applied to many computer vision task like classification, object detection, and segmentation. The specific network structure of ResNet-50 and ResNet-101 are shown in Table 1, where 7×7, 64, stride 2 stands for the convolution kernel size, number of filters, and convolution strides respectively, and 1000-d fc is a full-connect layers with 1000 units. In this paper, we take ResNet-101 as the backbone network.

### 2.2. RPN Subnetwork

The first stage of the two-stage detector is to generate candidate region proposal. Traditional region proposal methods such as Selective Search [25] and EdgeBoxes [26] are time-consuming and can not be trained end-to-end, Ren et al. [14] proposed a Region Proposal Network (RPN) to build a unified network. As a pre-detection stage, the RPN achieved an end-to-end object detection with the sharing convolution feature maps and realized the integration of classification and location. In this stage, region proposals are generated from the fusion layer, and these proposals are subsequently fed to the detection subnetwork for accurate classification and bounding box regression.

#### 2.2.1. Multilayer Fusion

A good detector should be able to detect objects with a large range of scale, hence FRCN uses high-level feature maps from the backbone network and computes the anchors on a single input scale to predict candidate bounding boxes with different scales and ratios. However, for the outputs of the backbone network, high-level feature maps have rich semantic information but they hardly have a response on small size objects, whereas low-level feature maps have higher resolution but semantic information are rare. In order to ease the inconsistency, inspired by [27,28], we combined high/low level layers to get a fusion layer, then region proposals are generated from the fusion layer with different filter sizes.

**Figure 3:**
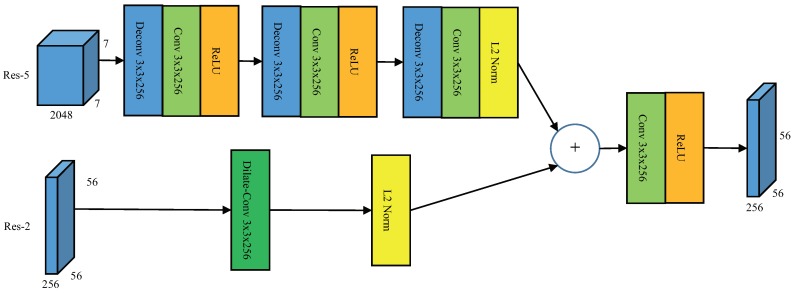
The structure of Multilayer fusion.

The multilayer fusion is shown in Figure 3, taking Res-2 and Res-5 as examples. In order to keep the fusion layer shape the same as Res-2, we need to upsample the spatial resolution of Res-5 by a factor of 8, which can be implement through 3 deconvolution layers with stride 2. The kernel size of deconvolution layers is 3×3 with 256 outputs. Following the deconvolution layer are plain 3×3 convolution, L2 normalization layers, and rectified linear unit (ReLU) activation layers. The outputs of Res-2 connect with a dilate convolution layer and L2 normalization layer, the aim of dilate convolution is to expand the receptive field, and the dilate is 2. The output fusion layer is achieved by element-wise summation of two branches after a convolution layer and a ReLU layer.

#### 2.2.2. Region Proposal Network

**Figure 4:**
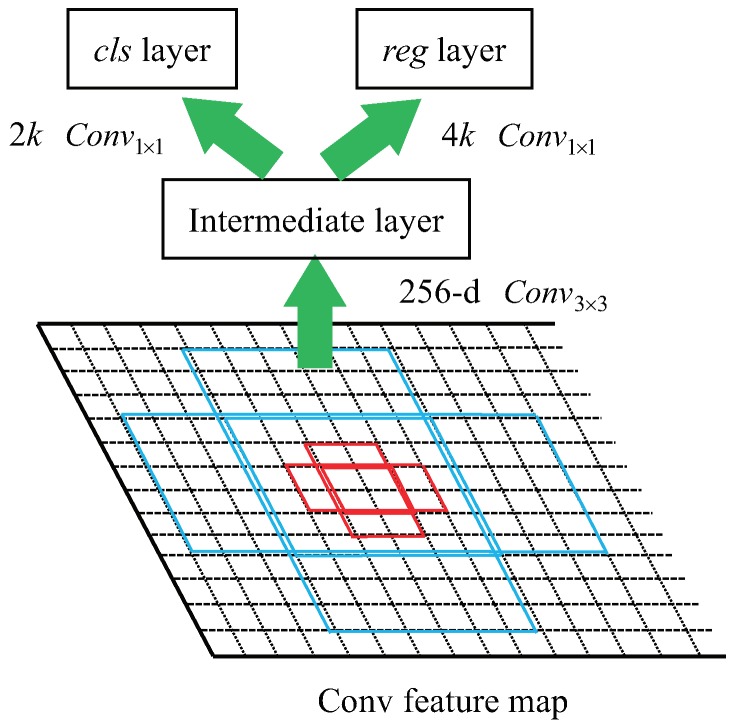
The structure of region proposal network. The red and blue retangles represent anchors with different scales and ratios.

The structure of RPN is shown in Figure 4, *k* region proposals, called anchors, are generated at each sliding-window location with different scales and ratios. After the multilayer fusion, the RPN is able to slide a fixed set of filters with multiscale receptive field over the feature maps, these anchor boxes are sent to a intermediate layer and mapped into a lower dimensional vector, then the intermediate layer is fed into two sibling layers for classification and regression. The regression layer has 4*k* outputs to encode the coordinates of anchors, and the classification layer has 2*k* outputs to estimate the probability of anchors being an object or not.

Since the region proposals are too large, and many proposals heavily overlap with each other, we apply non-maximum suppression (NMS) [29] to reduce the number of proposals. The judgement of whether the extracted region proposal is required depends on a metric of intersection-over-union (IOU), which is defined as follows:

(1) $$IOU=\frac{area\left(B_{i}\cap B_{i}^{*}\right)}{area\left(B_{i}\cup B_{i}^{*}\right)}$$

where $area\left(B_{i}\cap B_{i}^{*}\right)$ denotes the intersection of the proposal box and the ground truth box, and $area\left(B_{i}\cup B_{i}^{*}\right)$ denotes the union of those two parts. Anchors that have the highest IOU or have an IOU larger than 0.7 will be considered as a foreground region proposal and attribute with a positive label, and anchors that have IOU smaller than 0.3 will be considered as a background region proposal and attribute with a negative label. Anchors that are neither positive nor negative do not participate in training.

#### 2.2.3. Loss Function

With the above definitions, the multi-task loss function is a combination of classification and bounding box regression, which is defined as below:

(2) $$L\left(p_{i},t_{i}\right)=\frac{1}{N_{cls}}\sum_{i}L_{cls}\left(p_{i},p_{i}^{*}\right)+\lambda \frac{1}{N_{reg}}\sum_{i}p_{i}^{*}L_{reg}\left(t_{i},t_{i}^{*}\right),$$

where $p_{i}$ is the predicted probability of anchor *i* being an object, the ground truth label $p_{i}^{*}$ set as 1 if the anchor is positive, otherwise set as 0 if the anchor is negative. λ is a balancing weight for bounding box regression, $t_{i}$ is a vector representing the four parameterized coordinates of the predicted bounding box, and $t_{i}^{*}$ is that of the ground truth box associated with a positive anchor. For the regression loss, we use $L_{reg}\left(t_{i},t_{i}^{*}\right)=R\left(t_{i}-t_{i}^{*}\right)$, where R is a robust loss function defined as

(3) $$R\left(t_{i}-t_{i}^{*}\right)=\left\{\begin{array}{c} \begin{array}{cc} 0.5{\left(t_{i}-t_{i}^{*}\right)}^{2} & \left|t_{i}-t_{i}^{*}\right|<1 \end{array}\\ \begin{array}{cc} \left|t_{i}-t_{i}^{*}\right|-0.5 & others, \end{array} \end{array}\right.$$

The classification loss $L_{cls}$ is the log loss to judge an object or not. *Cross entropy* (CE) is the most popular loss function for object classification, taking the binary classification as example, the CE loss function is formally defined as:

(4) $$L_{CE}\left(p,y\right)=-\log\left(p_{t}\right),$$

with $p_{t}=\{\begin{array}{cc} p & if\;y=1\\ 1-p & otherwise \end{array}$, where $y\in \left\{\pm 1\right\}$ specifies the ground-truth class and $p\in \left[0,1\right]$ is the model’s estimated probability for the class with label y=1. CE loss can reduce the imbalance between positive and negative samples, but it is not good enough to train classifier for distinguishing easy and hard classified examples.

For the task of SAR ship detection, the objects near the shore are highly like the ships and can easily be falsely detected. To prevent the training from being dominated by easy examples and make the model more robust, focal loss ($L_{FL}$) [30] function is used to substitute for CE loss. With an adjustable parameter γ≥0, focal loss can be viewed as a factor ${\left(1-p_{t}\right)}^{\gamma }$ added to the CE loss, which is defined as follows:

(5) $$L_{FL}\left(p_{t}\right)=-{\left(1-p_{t}\right)}^{\gamma }\log\left(p_{t}\right).$$

**Figure 5:**
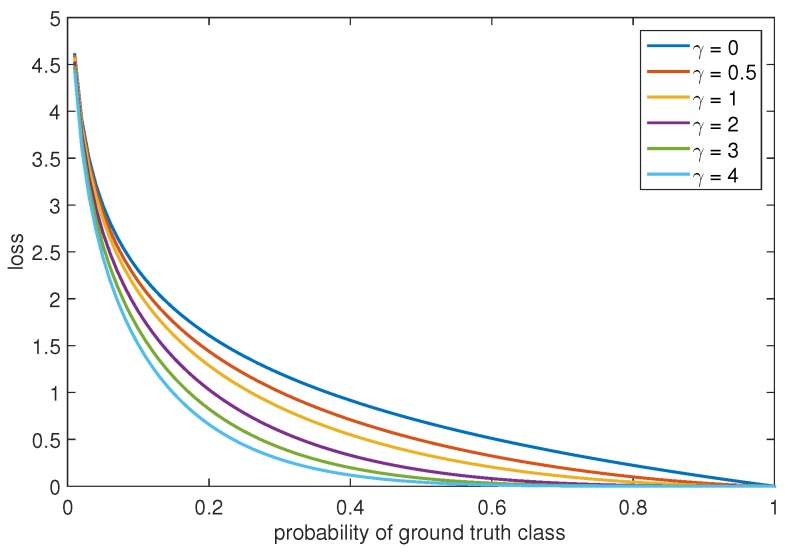
Focal loss curves for different values of γ.

As shown in Figure 5, the CE loss of well-classified examples ($p_{t}>0.5$) have a relatively large loss compared with focal loss. When an example is misclassified and $p_{t}$ is small, the modulating factor tends to 1 and the loss is unaffected. In contrast, when $p_{t}$ tends to 1, the modulating factor tends to 0, which down-weights the loss for well-classified examples. Specifically, focal loss degenerate into CE loss when γ=0. In summary, focal loss reduces the relative for well-classified examples and put more focus on hard, misclassified examples.

### 2.3. Detection Subnetwork

**Figure 6:**
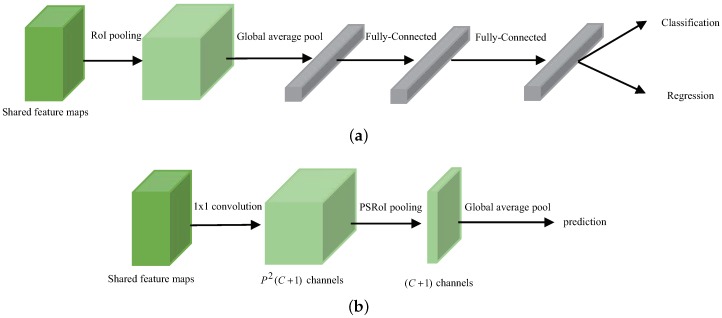
Two typical detection subnetwork. (**a**) FRCN detection subnetwork. (**b**) RFCN detection subnetwork.

The detection subnetwork is the second stage behind the RPN subnetwork to increase detection accuracy. It takes an image with coarse predicted region boxes as input and outputs the refined category and location simultaneously. FRCN and RFCN [31] are two typical two-stage detectors and the structures of their detection subnetwork are shown in Figure 6. The FRCN detection subnetwork adopts two large fully connected layers as the second stage classifier and achieves the leading accuracy in most tasks. However, the high dimension in fully connected layers will increase the computation, moreover, FRCN processes each RoI by loop, the computation could be intensive because the value of RoI is very large. To share the computation of RoI, RFCN expands the feature maps to $p^{2}\left(C+1\right)$ through 1×1 convolution, then adapts a position-sensitive pooling (PSRoI pooling) layer to pool along each RoI and average vote the final prediction. Generally speaking, there are several approaches to simplify the model complexity such as reducing the number of channels and reducing the number of layers. In the proposed method, we take advantage of the above two methods. Firstly, we replace plain convolution with a large-kernel separable convolution to produce a “thin” feature map. The number of channels, different from the RFCN subnetwork, depending on the number of classes, is a small fixed value. Then, we pool along each RoI and average vote the final prediction. Finally, a cheap single fully connected layer is attached to the pooling layer, which exploits the feature representation for classification and regression.

#### 2.3.1. Large-Kernel Separable Convolution

**Figure 7:**
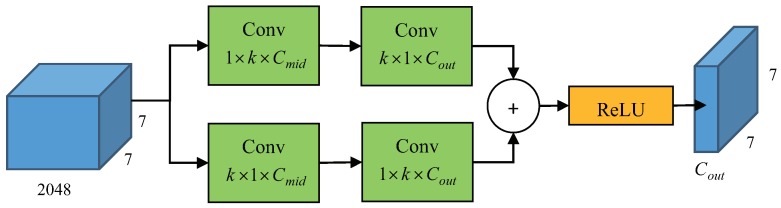
Large-kernel separable convolution performs a k×1 and 1×k convolution sequentially. The number of parameters can be controlled through alternating $C_{mid}$ and $C_{out}$.

In order to acquire a "thin" feature map, inspired by [32,33], large-kernel separable convolution is added to the fusion layer, the structure of which is shown in Figure 7. We simply extract the feature maps with different scale and contact them to get the output feature maps. In theory, the n×n convolution can be replaced by a 1×n convolution followed by a n×1 convolution, and this operation can keep the receptive field and save the computational budget as *n* grows. In our research, we set *k* as 15, $C_{mid}$ as 256 and $C_{out}=10\times p\times p$, where *p* is the pooling size of PSRoI. Benefiting from the separable convolution layers with valid receptive field, we can get a more powerful output feature map.

#### 2.3.2. Position-Sensitive RoI-Pooling

**Figure 8:**
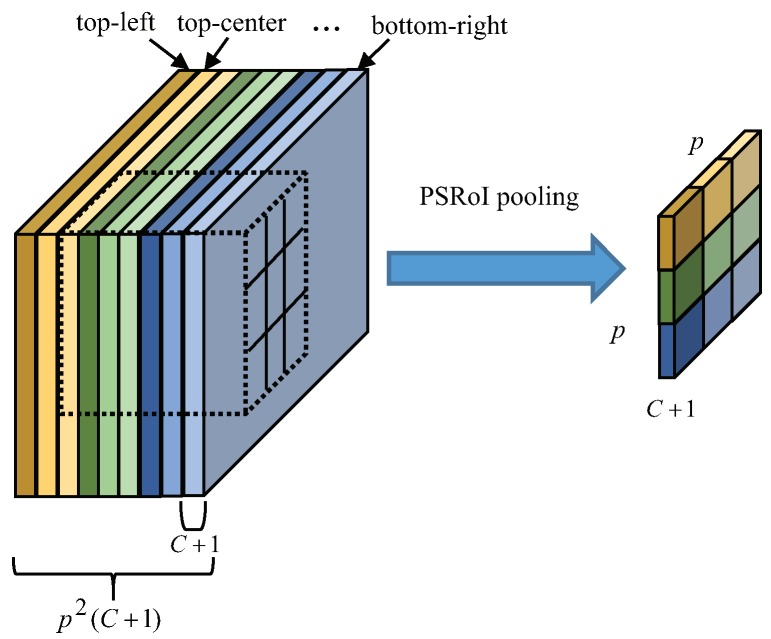
The architecture of position-sensitive region of interest (RoI)-pooling.

The imbalance of translation-invariance in the classification stage and translation-variance in the detection stage still exist in FRCN. Specifically, deeper convolution layers are less sensitive to translation, and classification task favors translation invariance. However, in the object detection task, the location information will become less sensitive with the increase network depth, which may cause inaccurate detection. Position-sensitive score maps [31] were proposed to address a dilemma between translation-invariance in the classification stage and translation-variance in the detection stage. As shown in Figure 8, with the position-sensitive RoI pooling layer, the last convolution layer produces a bank of p×p bins and generates a $p^{2}\left(C+1\right)$ channel output layer with *C* object categories (+1 for background).

## 3. Experiments and Results

In this section, we present the performance of the proposed method. Two experiments are designed to explore the effect of multilayer fusion and the influence of light-head design. In addition, the comparison with other methods indicates the outperformance of the proposed method.

### 3.1. Experimental Dataset and Settings

**Figure 9:**
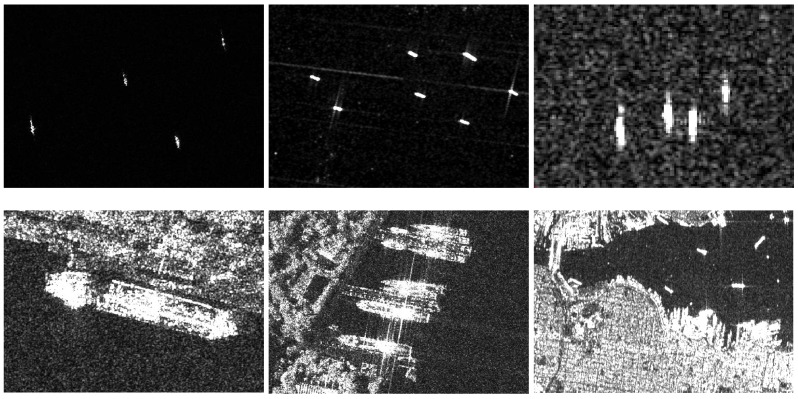
Samples from synthetic aperture radar (SAR) ship detection dataset (SSDD). The first line has ships in the open sea, the second line has ships near the dock and shore.

Following a similar format as PASCAL VOC [34], the public SAR Ship Detection Dataset (SSDD) [18], collected from Sentinel-1 RadarSat-2 and TerraSAR-X, has SAR images of different resolutions from 1 m to 15 m. The specific information of ships in SSDD is shown in Table 2. In SSDD, there 2456 ships in 1160 images in total, an average 2.12 ships per image. As some small ships only have very few pixels in low resolution, we would regard it as a ship and make the annotation if the number of pixels is more than three. Statistics for the number of ships and images are given in Table 3, where NoS is the abbreviation of number of ships, and NoI is the abbreviation for the number of images. We divide the dataset into three parts (training set, test set, and validation set) with the ratio of 7:2:1. Some examples of SSDD are shown in Figure 9.

#### 3.1.1. Experimental Settings

All experiments are implemented in the Tensorflow deep learning framework [35] and executed on a PC with a NVIDIA GTX1080 GPU. As is common practice, we use the pre-trained ResNet101 on the ImageNet dataset to initialize the model. During the training process, the images are rescaled with the shorter side as 600 pixels. For the anchors, we use 5 scales of $\left\{32^{2},64^{2},128^{2},256^{2},512^{2}\right\}$ and 3 aspect ratios of {1:1, 1:2, 2:1} to cover objects of different shapes, yielding 15 anchors at each sliding position. A mini-batch involves 1 images, 512 anchors, and 256 RoIs per image on GPU. We use a weight decay of 0.0001 and a momentum of 0.9. Each mini-batch has 2 images and each image has 2000 RoIs for training, batch normalization is also fixed for a faster experiment. The iterations of training are 50 k. The initial learning rate is 0.001 every 20 k decrease 10 times.

#### 3.1.2. Evaluation Indicators

To evaluate the quality of the model, the metrics of precision rate (*P*), recall rate (*R*), and $F_{1}$ score are defined as:

(6) $$P=\frac{TP}{TP+FP}$$

(7) $$R=\frac{TP}{TP+FN}$$

(8) $$F_{1}=\frac{2*P*R}{P+R}$$

where *TP*, *FN*, and *FP* denote the true positive, false negative, and false positive, respectively. Generally, if the area overlap ratio between the predicted bounding box and the ground-truth bounding box is larger than 0.5, the proposed detection map will be considered to be a *TP*; otherwise, it will be determined as a *FP*. Additionally, if several proposals overlap with the same ground-truth bounding box, only the one with maximum overlap is considered as a *TP*, and the others are considered as *FN*. The $F_{1}$ score is to evaluate the overall performance of detector, it reaches its best vale at 1 and worst at 0.

### 3.2. Ablation Study

#### 3.2.1. The Influence of Backbone Network

As mentioned before, the function of backbone network is to provide shared feature maps. To evaluate the influence of backbone network, we compare the ResNet with VGG-16. Both ResNet and VGG-16 are pre-trained from ImageNet, and multilayer fusion and focal loss are not applied in this experiment. As shown in Table 4, ResNet-101 achieves the best detection performance than others, and VGG-16 cost more time because it has the most weight parameters.

#### 3.2.2. The Influence of Multilayer Fusion

Following the above discussions, feature maps from different layers differ in terms of spatial resolution and semantic information. The low-level feature maps have high resolution but less semantic information, whereas the high-level feature maps have low resolution but more semantic information. Therefore, layer selection has a great impact on the performance of the detection system.

To identify the effect of multilayer fusion, comparison experiments with three different fusion strategies are conducted in this section. Specifically, model 1 combines the output of Res-1 and Res-5 for region proposal, model 2 combines Res-1 and Res-4, and model 3 integrate Res-2 and Res-5. The baseline method is a model with a single layer Res-5. All models have the same detection subnetwrok as the proposed method.

**Figure 10:**
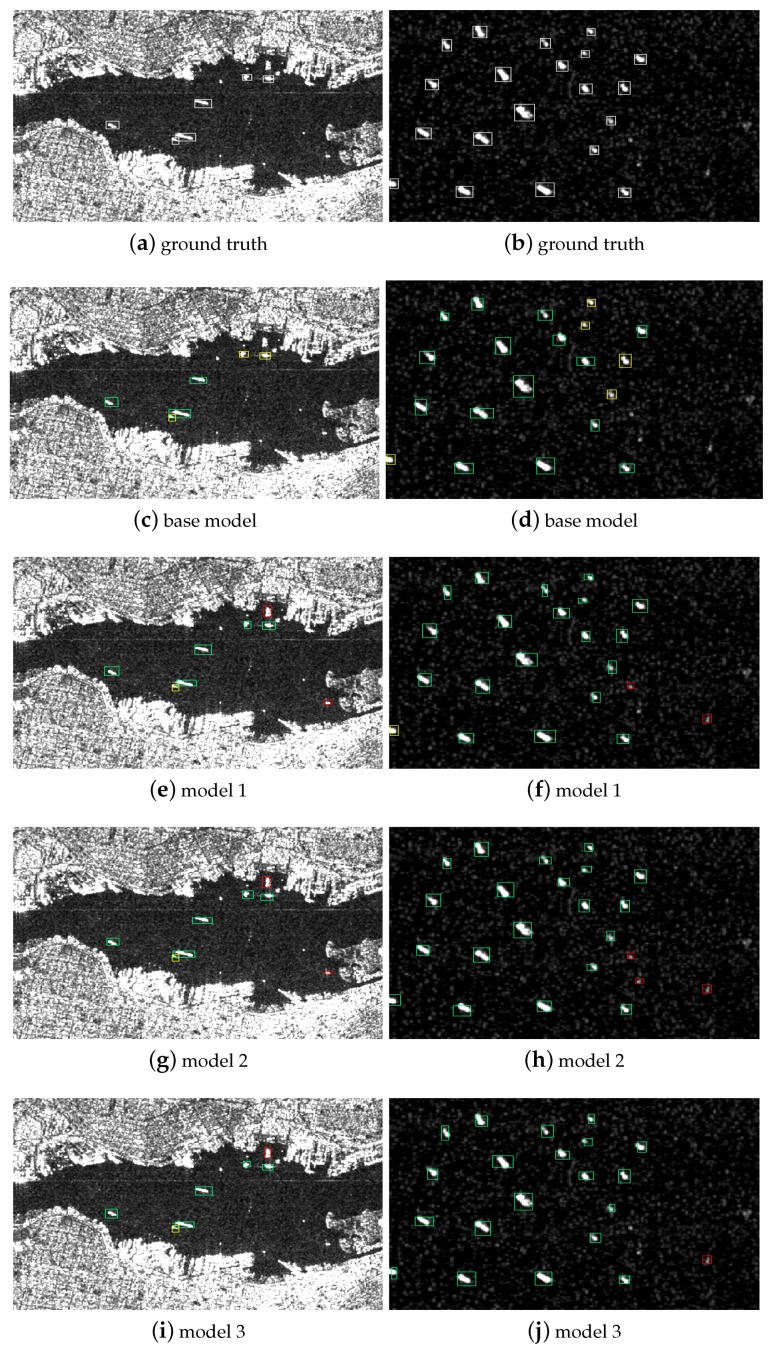
Detection results with different fusion layers. The left row is the SAR image of ships near the shore, the right row is the SAR image of ships in the open sea. The green, red, and yellow rectangles represent the positive detection, false detection, and missing ships, respectively.

Figure 10 shows the test result of different model, the left row is the SAR image near the shore, the right row is SAR image in the open sea. Generally speaking, the base model connected to a single layer omits several small and densely packed objects, multilayer fusion shows superior performance in multiscale object detection. In model 2 and model 3, several negetive samples are treated as targets, and model 4 achieves the best result for it can detect multiscale objects and make the fewest false detections.

Table 5 displays the detection probability, false alarm probability, and $F_{1}$ scores of different layer fusion strategies. In summary, compared with the performance on base model, the network with multilayer fusion achieve superior performance on both evaluation indicators. Specifically, model 1 with the fusion layer Res-1 and Res-5 obtains the lowest false alarm probability, model 2 with the fusion layer Res-2 and Res-5 shows the best performance on both detection probability and $F_{1}$ score.

#### 3.2.3. The Influence of Parameter γ in Focal loss

In order to identify the influence of adjustable parameter in focal loss, comparison experiments with different values of γ in focal loss are conducted in this section. The scope of γ is {0,0.5,1,2,3,4}, when γ=0, focal loss is equivalent to CE loss. The combination strategy of Res-2 and Res-5 is adopted, and all models have the same experiment settings.

Table 6 shows the performance of models with different γ. It is obvious that focal loss has a better performance than CE loss in detection accuracy. The model γ=3 has the best result in precision rate and recall rate.

### 3.3. Comparison with Other Methods

#### 3.3.1. Experiments on SSDD

To validate the performance of our proposed method, we compare the proposed method with two-stage detector FRCN and one-stage detector SSD, and the settings of FRCN and SSD are the same as they proposed. As shown in Table 7, one stage detector SSD has the fastest detection speed, but its detection accuracy is not good for it does not have a region proposal stage. In terms of detection accuracy, due to the multilayer fusion structure of shallow and deep layer, the proposed method achieves superior performance than FRCN and SSD. In general, the proposed method greatly improves the detection accuracy without losing too much detection speed.

#### 3.3.2. Experiments on Sentinel-1 Images

**Figure 11:**
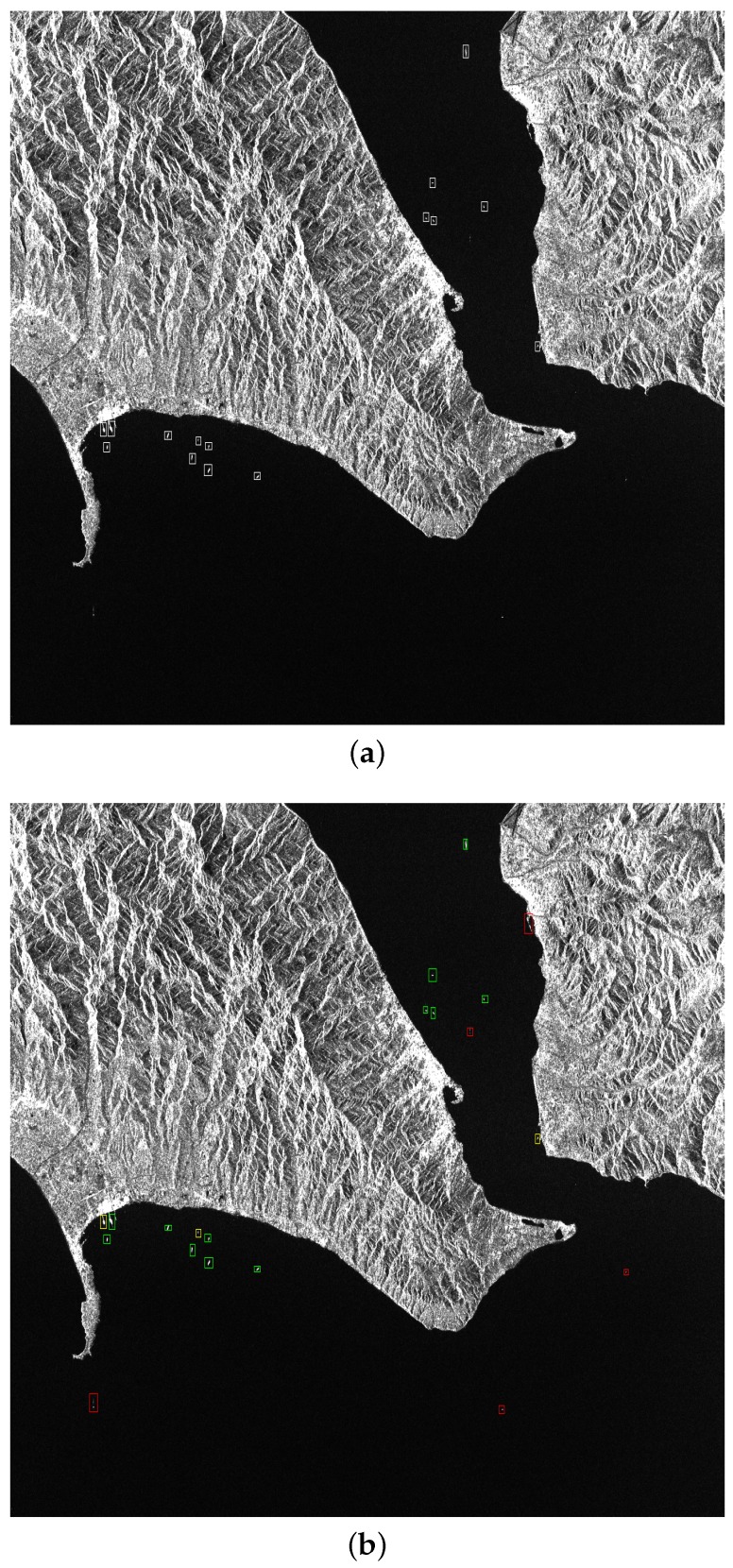

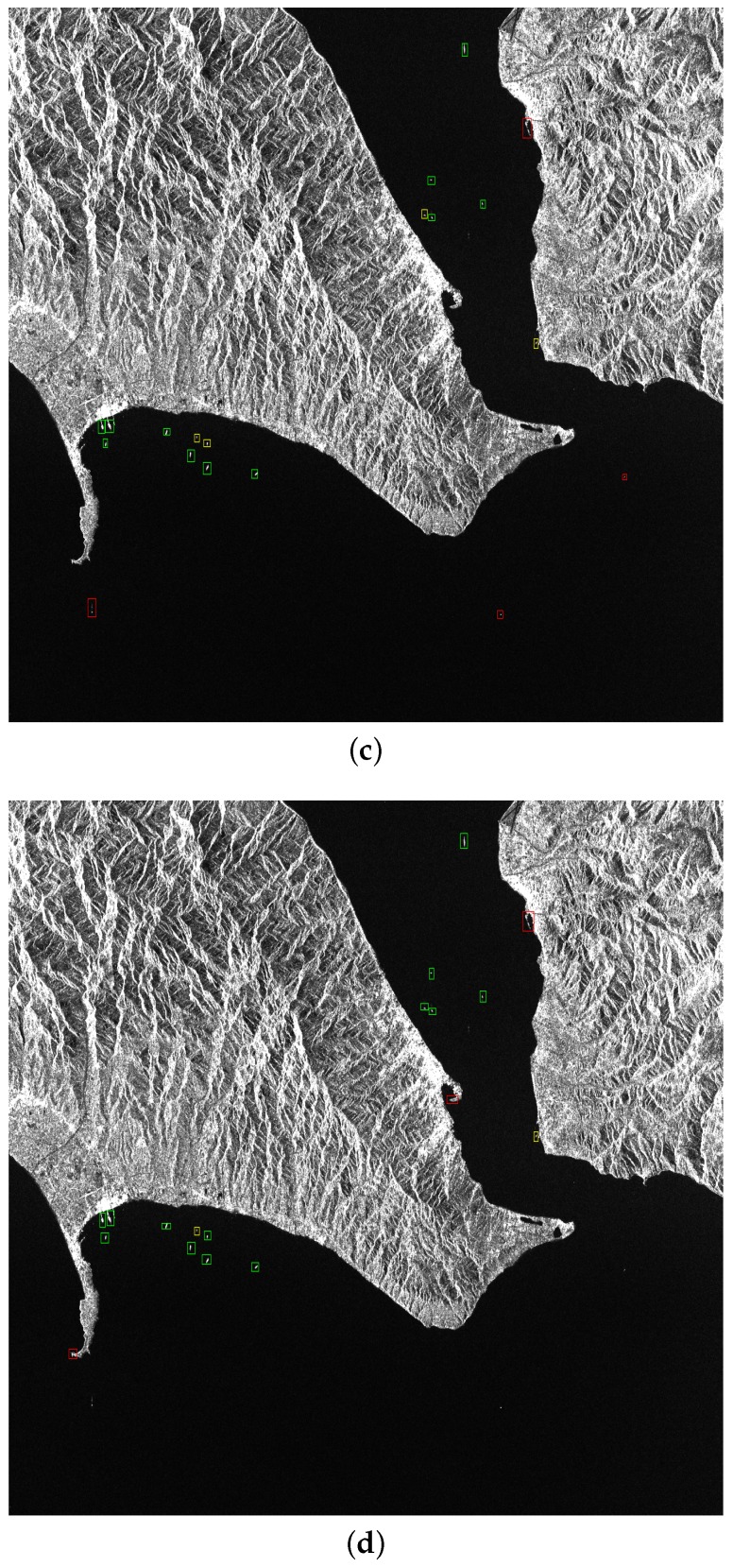
The detection result of Sentinel-1 image. (**a**) Ground truth (**b**) FC-constant false alarm rate (CFAR) (**c**) IS-CFAR (**d**) proposed. The white, green, red, and yellow rectangles represent the ground truth, positive detection, false detection, and missing ships, respectively.

In this experiment, we compare the proposed method with statistically based IS-CFAR [7] and physically based FC-CFAR [10]. Both of the CFAR methods are under the Gaussian distribution assumption and false alarm probability is set as $P_{f}a=10^{-6}$. The test SAR image, a harbor in England with ground resolution approximately 10 m, was provided by the European Space Agency (ESA) with 1313×907 pixels. The polarization mode is HV and the scanning mode is stripMap. It is manually determined that there are 15 target ships. The detection results are shown in Figure 11 and Table 8. In terms of detection performance, our proposed method has superior ability to detect ships near the shore and CFAR-based methods are more sensitive to detect ships in the open sea. The reason is that the deep CNNs are based on feature representation and CFARs are based on pixel distribution. Additionally, our proposed method is a unified end-to-end framework and has a speed advantage compared with the CFAR-based method.

## 4. Conclusions

In this paper, we propose a multilayer fusion light-head detector (MFLHD) for SAR ship detection. In order to detect multiscale ships, shallow and deep layers are combined to obtain high-resolution and semantic feature maps. In the detection subnetwork, light-head detector combined large-kernel separable convolution and position sensitive pooling is added to improve the detection speed. Additionally, for the imbalance of easy and hard examples, we adopt a focal loss function instead of cross entropy to reduce the effect of easy examples during the training process. Experiments conducted in this paper validate the superior performance both in detection accuracy and speed.

## Figures and Tables

**Table 1:** The specific structure of ResNet-50 and ResNet-101.

Layer Name	Res-1	Res-2		Res-3	Res-4	Res-5	Others
ResNet-50	7×7, 64 stride 2	3×3 maxpooling stride 2	$\left[\begin{array}{c} 1\times 1,64\\ 3\times 3,64\\ 1\times 1,256 \end{array}\right]\times 3$	$\left[\begin{array}{c} 1\times 1,128\\ 3\times 3,128\\ 1\times 1,512 \end{array}\right]\times 4$	$\left[\begin{array}{c} 1\times 1,256\\ 3\times 3,256\\ 1\times 1,1024 \end{array}\right]\times 6$	$\left[\begin{array}{c} 1\times 1,512\\ 3\times 3,512\\ 1\times 1,2048 \end{array}\right]\times 3$	average pooling 1000-d fc softmax
ResNet-101					$\left[\begin{array}{c} 1\times 1,512\\ 3\times 3,512\\ 1\times 1,2048 \end{array}\right]\times 23$		

**Table 2:** The synthetic aperture radar (SAR) ship detection dataset (SSDD) contains different kinds of SAR ship image.

Sensors	Polarization	Scale	Ship	Resolution	Position
Sentinel-1 RadarSat-2 TerraSAR-X	HH, VV VH, HV	1:1 1:2 2:1	Different size and material	1–15 m	in the sea and offshore

**Table 3:** The detailed information of SSDD.

NoS	1	2	3	4	5	6	7	8	9	10	11	12	13
**NoI**	725	183	89	47	45	16	15	8	4	11	5	3	3

**Table 4:** Detection performance with different backbone network.

Backbone Network	Precision Rate	Recall Rate	$F_{1}$	Testing Time (ms)
ResNet-50	76.5%	71.8%	0.741	88
ResNet-101	77.4%	73.6%	0.755	92
VGG-16	76.8%	71.4%	0.740	168

**Table 5:** Detection performance with different layer fusion strategies.

Models	Strategy	Precision Rate	Recall Rate	$F_{1}$
base model	Res-5	83.4%	75.0%	0.802
model 1	Res-1 + Res-5	85.2%	**82.0%**	0.836
model 2	Res-1 + Res-4	84.6%	80.4%	0.824
model 3	Res-2 + Res-5	**87.5%**	81.6%	**0.844**

**Table 6:** Detection performance of the model with different γ.

γ	Precision Rate	Recall Rate	$F_{1}$
0	84.6%	80.7%	0.826
0.5	84.8%	79.5%	0.821
1	85.9%	80.3%	0.830
2	86.2%	**82.2%**	0.841
3	**87.5%**	81.6%	**0.844**
4	85.7%	80.3%	0.829

**Table 7:** Detection performance comparison between three methods.

Method	Precision Rate	Recall Rate	$F_{1}$	Testing Time (ms)
FRCN	82.3%	73.2%	0.766	228
SSD	72.4%	68.7%	0.705	**82**
Proposed method	**87.5%**	**81.6%**	**0.844**	102

**Table 8:** Detection performance comparison between our method and the reference methods on Sentinel-1 image.

Method	Number of Detected Ship Targets	Number of True Positive	Number of True Negetive	Number of False Positive	Testing Time
FC-CFAR	19	11	4	4	228 s
IS-CFAR	20	12	3	5	286 s
Proposed method	18	13	2	3	3.4 s
